# Early Diagnosis of Latent Tuberculosis Reactivation due to Drug Interaction between Cobicistat and Intranasal Fluticasone

**DOI:** 10.1155/2019/8243868

**Published:** 2019-12-03

**Authors:** Roberto Pineda-Reyes, Alena Klochko

**Affiliations:** ^1^Department of Internal Medicine, University of Central Florida College of Medicine, Orlando, FL, USA; ^2^Infectious Disease Division, Orlando Veterans Affairs Medical Center, Orlando, FL, USA

## Abstract

**Background:**

Single-tablet antiretroviral therapy is currently the first-line choice for the treatment of HIV infection. Some therapeutic regimens contain the CYP3A4 inhibitor cobicistat, which can interact with drugs undergoing hepatic first-pass metabolism, leading to unintended adverse effects.

**Case Presentation:**

A 41-year-old man presented to the HIV clinic following a visit to the Emergency Department. His CD4^+^ count was 1,271 cells/*μ*L, and viral load was undetectable in the previous month. The patient was on an antiretroviral therapy regimen containing cobicistat. He reported using a self-initiated over-the-counter fluticasone nasal spray for at least 2 weeks prior. He had a history of positive tuberculin skin test and a negative chest X-ray within the past year. He denied cough and was in no respiratory distress. A chest CT scan revealed a new thick-walled cavitary nodule in the right upper lobe. A CT-guided biopsy of the lesion yielded *Mycobacterium tuberculosis*.

**Conclusions:**

HIV-infected individuals have higher risk for tuberculosis reactivation regardless of their CD4^+^ count. Fluticasone's hepatic metabolism is bypassed in the presence of CYP3A4 inhibitors, which increases its systemic bioavailability and the risk for impaired immunity. The goal of this report is to increase awareness among physicians about the potential adverse outcomes from the interaction of these drugs.

## 1. Introduction

The highly active antiretroviral therapy (HAART) regimen composed of elvitegravir/cobicistat/emtricitabine/tenofovir alafenamide (EVG/c/FTC/TAF) is one of the recommended first-line choices for the treatment of the human immunodeficiency virus (HIV) infection [1], being popular among other reasons, due to its daily single-tablet formulation which facilitates medication adherence. One of its constituents, cobicistat, is a pharmaco-enhancer that acts as a potent inhibitor of the cytochrome P450 3A4 (CYP3A4), boosting the action of EVG [2]. This pharmacokinetic feature raises concerns for interactions with other drugs metabolized through the same pathway, as their plasma concentration could be potentially increased.

Intranasal and inhaled corticosteroids (ICS), particularly fluticasone, are well-known substrates of CYP3A4, and their interaction with cobicistat has been linked to steroid-induced adverse effects including Cushing's syndrome and adrenal insufficiency [2–4]. Yet, immunosuppressive effects as an unintended result of this interaction, such as reactivation of latent tuberculosis infection (LTBI), have not been documented.

Here, we present the unique case of what we believe is the interaction of cobicistat with intranasal fluticasone in an otherwise well-controlled HIV-infected individual, leading to immune system impairment, esophageal candidiasis, and ultimately reactivation of LTBI. Even though the risk for opportunistic infections and reactivation of LTBI among subjects using ICS is thought to be dose-dependent, a significantly increased risk has been reported with the use of large doses [5, 6]. Additionally, HIV-infected subjects are at higher risk for LTBI reactivation compared with the non-HIV population regardless of the T-lymphocyte cluster of differentiation 4 (CD4^+^) count [7]. Thus, we postulate that the use of intranasal fluticasone concomitantly with the CYP3A4 inhibitor cobicistat may have rendered this patient susceptible to reactivation of LTBI.

## 2. Case Presentation

**Figure 1:**
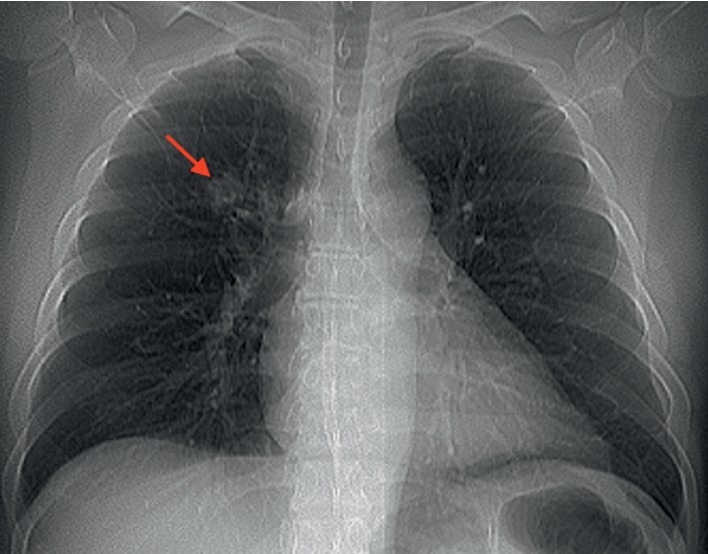
Chest X-ray showing a new right upper lobe nodule (red arrow).

A 41-year-old man presented to the HIV clinic for a follow-up after a recent visit to the Emergency Department at another hospital for dysphagia. His medical history was significant for HIV infection with last known CD4^+^ count 1,271 cells/*μ*L and undetectable viral load within the previous month. The patient was on HAART with EVG/c/FTC/TAF with adequate adherence for the past two years. He stated that his dysphagia progressed over 2 weeks, and it was initially associated with hiccups and then odynophagia and white pharyngeal plaques. In the Emergency Department, his laboratory exams showed a white blood cell count of 18 × 10^3^/*μ*L with monocyte predominance, and a chest X-ray revealed a new 2 cm right upper lobe infiltrate with nodular characteristics (Figure 1). The patient had normal vital signs and lacked respiratory symptoms. He was diagnosed with esophageal candidiasis, prescribed oral fluconazole, and was discharged home with pending outpatient follow-up of the abovementioned radiological findings. Of note, the patient did not disclose his HIV status during the encounter.

**Figure 2:**
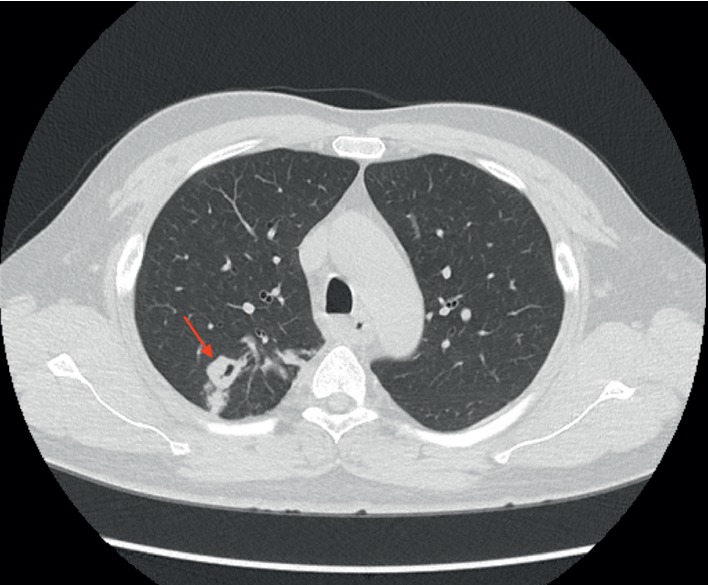
Chest CT scan revealing a new thick-walled cavitary nodule in the right upper lobe (red arrow).

The next day, upon evaluation at the clinic, the patient denied cough, night sweats, fever or chills, but reported noticing a 5-pound unintentional weight loss, which he attributed to decreased appetite and swallowing difficulties. In addition, he reported using a self-initiated over-the-counter fluticasone nasal spray 2-3 times per day for at least 2 weeks prior to presentation. Other past medical history included essential hypertension and dyslipidemia. The patient had a documented history of positive tuberculin skin test and had completed 6 months of treatment with isoniazid 21 years prior to this presentation. He had a negative chest X-ray within the past year. He had a family history of type-2 diabetes. Social history was significant for trips to visit his family in Philippines and Mexico during the previous year and daily tobacco smoking for 17 years. On exam, he was not in respiratory distress and had mild posterior pharyngeal and nasal erythema. The rest of the physical examination was noncontributory. Given the abnormal X-ray findings and the suspicion for immunosuppression based on the presence of esophageal candidiasis, the patient was immediately placed on airborne precautions and a chest computed tomography (CT) scan was ordered, which revealed a new thick-walled right upper lobe cavitary nodule measuring 19 × 14 mm (Figure 2). The patient was admitted to the inpatient ward for further evaluation. Bronchoalveolar lavage (BAL), acid-fast bacillary (AFB) smear, and cultures were all negative. Serology for *Aspergillus*, *Blastomyces*, *Histoplasma*, and *Cryptococcus* were negative. Polymerase chain reaction in serum for cytomegalovirus was negative. A CT-guided lung biopsy of the nodular lesion was performed, and the AFB culture yielded *Mycobacterium tuberculosis*. Therefore, the decision was made to start antituberculosis therapy with rifabutin, isoniazid, pyrazinamide, and ethambutol, and the HAART regimen was switched to efavirenz/emtricitabine/tenofovir disoproxil fumarate for a better drug interaction profile. Rifabutin was prescribed at an oral daily dose of 450 mg due to interaction with efavirenz. The patient was discharged in stable condition and currently has regular follow-ups in our HIV clinic.

## 3. Discussion

To the best of our knowledge, this is the first report that associates the interaction between cobicistat and fluticasone with important infectious consequences such as the reactivation of LTBI.

The newest available intranasal corticosteroids, such as fluticasone propionate or mometasone furoate, exhibit pharmacokinetic and pharmacodynamic properties that enhance their potency, while also allowing them to have significantly less systemic absorption compared with the older agents, in which up to one-third of the administered dose may reach the systemic circulation [8]. These properties include: (1) the affinity to the glucocorticoid receptor (RRA: relative receptor-binding affinity); (2) the lipophilicity, which provides faster absorption by the nasal mucosa and longer retention in the local tissue; and (3) the degree of systemic absorption and bioavailability [8]. Thus, an ideal compound would have a high RRA, high lipophilicity, and low systemic absorption. In the case of fluticasone propionate, the latter characteristic is due to both the high lipophilicity of the propionate ester side chain and the extensive first-pass metabolism through the CYP3A4 [9].

Theoretically, upon intranasal administration of the drug, up to 70% of it can be swallowed and available for systemic absorption through the gastrointestinal mucosa, which will ultimately undergo hepatic first-pass metabolism. Under normal circumstances, about 99% would be metabolized by CYP3A4, making the fraction of systemically absorbed drug negligible [8].

The bypass of hepatic metabolism by intranasal or ICS, particularly fluticasone, due to inhibition of the CYP3A4 by protease inhibitors or by cobicistat, has been extensively discussed in the literature. Several reports demonstrate that this interaction can lead to systemic steroid accumulation, suppression of the hypothalamic-pituitary-adrenal axis, and secondary Cushing's syndrome as well as adrenal insufficiency [2–4, 10].

Corticosteroids impact the immune system through numerous pathways. They induce neutrophil and monocyte dysfunction, decrease the release of interleukin-1 and tumor necrosis factor (TNF)-alpha, and also may inhibit critical macrophage antimycobacterial machinery such as autophagy induction and nitric oxide production [6, 11, 12]. The risk for any infection is known to be dependent on the dose of corticosteroids, and doses as low as the equivalent of prednisone 7.5 mg/day have been associated with increased risk [13]. Moreover, with the advent and popularization of nasal and ICS, there is growing evidence of their association with tuberculosis, especially since 1,000 *μ*g of fluticasone propionate is estimated to be equivalent to 10 mg of prednisone [6]. This was demonstrated in two studies by Lee et al. [6] and Brassard et al. [5], who found that in the absence of oral corticosteroid therapy, subjects on ICS had almost double the risk for developing tuberculosis when using doses of 1,000 *μ*g/day of fluticasone. It is worth mentioning that studies specifically evaluating intranasal corticosteroids as a risk factor for tuberculosis are lacking likely due to their relatively low doses and pharmacokinetic features. However, careful use of both intranasal and ICS is recommended in the presence of CYP3A4 inhibitors given the risk of iatrogenic Cushing's syndrome and other complications [14].

Among HIV-infected individuals, both the risk for tuberculosis infection and the risk for progression from LTBI to active disease are higher when compared to the general population regardless of the CD4^+^ count [7]. Although the mechanisms are not well understood, theories including HIV-induced macrophage dysfunction, reduced nitric oxide synthase activity, poorly formed granulomas, and decreased TNF-alpha release have been proposed [15, 16].

Our patient had some well-known risk factors for reactivation of LTBI: HIV infection, chronic tobacco smoking, and corticosteroid use [7, 17]. Interestingly, he presented with esophageal candidiasis, which according to the literature is much more prevalent among subjects with low CD4^+^ counts, in those who are not on HAART, or in those treated with high doses of ICS [18, 19]. These facts raised the question of why a well-controlled patient with normal CD4^+^ count, without history of sick contacts, and living in a low-risk country for tuberculosis, would develop esophageal candidiasis and ultimately progression of LTBI to active disease. We believe that the unsupervised use of intranasal corticosteroids, while on the CYP3A4 inhibitor cobicistat, rendered this patient susceptible to reactivation of LTBI. Using the drug interaction probability scale (DIPS) we calculated a score of 5, which qualifies as “probable” for this drug-drug interaction [20]. Notably, given the absence of respiratory symptoms on presentation, as well as negative sputum and BAL AFB studies, we consider we were fortunately able to catch an incipient form of disease.

Furthermore, it is important to acknowledge that the studies that demonstrated the association between ICS and tuberculosis considered significant at least one-month duration therapy. However, cases of LTBI reactivation have been described with much shorter courses of supraphysiological doses of steroids [21]. On the other hand, biochemical evidence of secondary adrenal suppression due to exogenous glucocorticoid exposure would have been ideal in order to support our hypothesis, but unfortunately these tests were not obtained upon patient's admission.

Intranasal and ICS must be carefully selected in patients receiving CYP3A4 inhibitors, and other drugs or steroid-sparing interventions should be considered accordingly. Patients taking cobicistat or protease inhibitors must be extensively educated and emphasized on the importance of these drug interactions. A suggested alternative is triamcinolone for nasal use and beclomethasone for inhaled use. Although they are still substrates of CYP3A4, these drugs have additional metabolic routes such as lung esterases and other subsets of the cytochrome P450 [10, 22]. Otherwise, an alternative HAART regimen avoiding the cobicistat component should be chosen for the treatment of the HIV infection.

## 4. Conclusion

In summary, the goal of this report was to increase awareness in the medical community about the potential consequences of this drug interaction. Specifically, it is important to know the various drugs in combination therapies for treatment of HIV, which are frequently referred to by their single-word brand names. Physicians should perform a meticulous and accurate medication reconciliation that includes the ones obtained over-the-counter and also to educate their patients about the potential side effects and interactions of these drugs.

## Abbreviations

HAART: Highly active antiretroviral therapy
EVG/c/FTC/TAF: Elvitegravir/cobicistat/emtricitabine/tenofovir alafenamide
HIV: Human immunodeficiency virus
CYP3A4: Cytochrome P450 3A4
ICS: Inhaled corticosteroids
LTBI: Latent tuberculosis infection
CD4^+^: T-lymphocyte cluster of differentiation 4
CT: Computed tomography
BAL: Bronchoalveolar lavage
AFB: Acid-fast bacillary
RRA: Relative receptor-binding affinity
TNF: Tumor necrosis factor.
